# Emerging role of BMPs/BMPR2 signaling pathway in treatment for pulmonary fibrosis

**DOI:** 10.1016/j.biopha.2024.117178

**Published:** 2024-08-13

**Authors:** Qinmao Ye, Sarah J. Taleb, Jing Zhao, Yutong Zhao

**Affiliations:** a Department of Physiology and Cell Biology, Dorothy M. Davis Heart and Lung Research Institute, United States; b Department of internal Medicine, the Ohio State University, Columbus, OH, United States

**Keywords:** Pulmonary fibrosis, TGF-β pathway, BMP ligands, BMP receptors, Myofibroblast differentiation, Pro-fibrotic responses, Anti-fibrosis

## Abstract

Pulmonary fibrosis is a fatal and chronic lung disease that is characterized by accumulation of thickened scar in the lungs and impairment of gas exchange. The cases with unknown etiology are referred as idiopathic pulmonary fibrosis (IPF). There are currently no effective therapeutics to cure the disease; thus, the investigation of the pathogenesis of IPF is of great importance. Recent studies on bone morphogenic proteins (BMPs) and their receptors have indicated that reduction of BMP signaling in lungs may play a significant role in the development of lung fibrosis. BMPs are members of TGF-β superfamily, and they have been shown to play an anti-fibrotic role in combating TGF-β-mediated pathways. The impact of BMP receptors, in particular BMPR2, on pulmonary fibrosis is growing attraction to researchers. Previous studies on BMPR2 have often focused on pulmonary arterial hypertension (PAH). Given the strong clinical association between PAH and lung fibrosis, understanding BMPs/BMPR2-mediated signaling pathway is important for development of therapeutic strategies to treat IPF. In this review, we comprehensively review recent studies regarding the biological functions of BMPs and their receptors in lungs, especially focusing on their roles in the pathogenesis of pulmonary fibrosis and fibrosis resolution.

## 1. Introduction

Pulmonary fibrosis (PF) is a chronic, progressive, and irreversible lung disease. Fibrosis in lung tissue is initiated by abnormal lung repair processes followed by recurrent lung inflammatory injuries [1,2]. PF results in thickening and hardening of the lung tissues, interfering with gas exchange. Some cases of PF show no clear origins or causes and are referred as idiopathic pulmonary fibrosis (IPF). PF is characterized by aberrant myofibroblast differentiation and extracellular matrix (ECM) accumulation [3]. The estimated median survival time of IPF is 3–5 years, with a high incidence and mortality in the elderly population. Currently, there are two FDA-approved drugs, pirfenidone and nintedanib, for IPF treatment; however, these two drugs only reduce the progression of PF in patients and can not reverse fibrosis or cure this disease. Lung transplantation is the ultimate therapy for IPF patients [3,4]. Due to the lack of therapeutic agents for IPF, understanding its pathogenesis is extremely important.

Bone morphogenetic proteins (BMPs) belong to the transforming growth factor beta (TGF-β) family [5]. In the BMPs’ canonical pathway, BMPs initiate a signal transduction cascade by binding to cell surface receptors (BMP receptor type I and type II) to induce cellular responses [6–8]. The anti-fibrotic effects of BMPs have been reported in different organs including lungs [9–12]. BMPR2 is a commonly expressed transmembrane serine/threonine receptor of the TGF-β superfamily [13,14]. Activation of BMPR2 by BMPs is thought to play a suppressed role against TGF-β signaling [15].

BMPR2 has been demonstrated to be strongly associated with pulmonary arterial hypertension (PAH), a progressive disease characterized by elevated pressure in the pulmonary artery and right ventricle hypertrophy. Approximately 70 % of inherited PAH patients and 25 % of idiopathic PAH (iPAH) patients have been found to have *BMPR2* gene mutations [16,17]. Recent studies indicate that BMPR2 is not only associated with PAH but also possibly involved in the pathogenesis of PF [18]. Therefore, this review will focus on the recent findings on the impact of BMPs and BMPR2 in PF.

## 2. The pathogenesis of PF

**Fig. 1.**
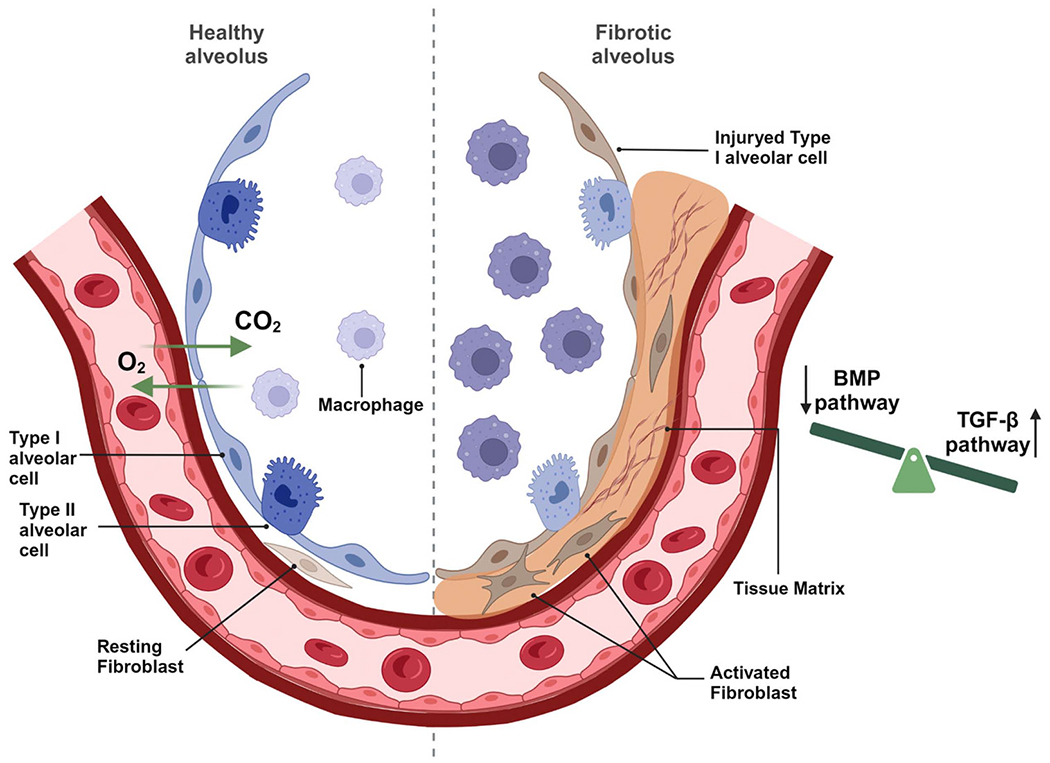
Pathogenesis of pulmonary fibrosis. Alveolar inflammation and injury induce fibroblast activation and differentiation to myofibroblast and ECM accumulation. Activation of TGF-β pathway and reduced BMP pathway contribute to the pathogenesis of PF.

PF is a chronic and progressive fibrotic lung disease that occurs when lung tissues become damaged and scarred [2–4]. Patients of PF generally exhibit shortness of breath, dry cough, and fatigue. Honeycombing changes in lungs can be observed by computed tomography (CT) scans [4,19]. The scarring associated with PF is initiated by repetitive alveolar injuries. Under ordinary circumstances, the lungs undergo a series of self-repair processes after injury. Upon tissue injury, cytokines and chemokines are released to recruit immune cells, such as neutrophils and macrophages, to combat infections and clean injured cells and debris. Immune cells also produce profibrotic factors, including TGF-β1, platelet derived growth factor (PDGF), fibroblast growth factor (FGF), and other factors to activate fibroblast cells and induce their differentiation into myofibroblasts to promote wound healing. Myofibroblasts produce extracellular matrix (ECM) to maintain the integrity of the injured tissue [20,21] (Fig. 1). In successful tissue repair, the ECM is degraded, and the myofibroblasts undergo apoptosis or dedifferentiation to inactive cell types [22–24]. However, abnormal myofibroblast differentiation and ECM accumulation in interstitial lung tissues cause progressive thickened and stiff tissues, ultimately interfering with gas exchange.

## 3. Resolution strategies of PF

Three steps to promote fibrosis resolution in the treatment of PF haven been considered [25]. Briefly, the first step is to determine the initial cause of injury, followed by application of the appropriate treatment to prevent further damage [26,27]. However, the exact causes of lung injury are often not identified in the onset of IPF. The next important step is the removal of the large amount of ECM deposition in the scar area of lungs during the progression of PF [25]. Finally, the most crucial step is the elimination or inactivation of fibrotic myofibroblasts [21,22]. Myofibroblast differentiation is generally considered a critical step in the development of fibrosis [28]. The markers of myofibroblast differentiation are increased α-smooth muscle actin (α-SMA) levels and subsequent ECM protein production, such as collagen and fibronectin (FN). If the transition and proliferation of myofibroblasts are not interrupted in the course of fibrosis resolution, the ECM accumulation will continue to occur and the PF cannot be reversed. Activated myofibroblasts are generally derived from at least three different sources: 1) resident fibroblasts, 2) epithelial and endothelial cells that undergo epithelial-mesenchymal transition (EMT) and endothelial-to-mesenchymal (EndMT) transition, and 3) circulating progenitors [29–32]. The roles of EMT and EndMT in PF are debatable due to lack supporting evidence from clinical histological analysis. BMPs and BMPR2 are involved in fibrosis resolution, and the next section will focus on their impact and possible therapeutic roles in this context.

## 4. BMPs and BMPR2 canonical pathways

BMPs are the members of TGF-β family [13,14]. There are more than 60 TGF-β superfamily members that have been identified and involved in a wide range of cellular processes [33]. TGF-β superfamily members are classified into TGF-βs, activins, inhibins, BMPs, growth and differentiation factors (GDFs) [34]. In canonical signaling pathways, TGF-β(1/2) or BMP proteins induce phosphorylation of intracellular downstream targets (Smads) to transduce TGF-β/BMP signals. In the TGF-β-mediated pathway, Smad2/3 are phosphorylated by TGF-β receptors (I/II) and combine with Smad4 to regulate expression of target genes (α-SMA and ECM proteins, such as fibronectin and collagens) [35]. BMPs were originally identified as factors that regulate bone formation and development, although it has been reported that not all BMP members demonstrate osteogenic properties [36,37]. Based on their structures and functions, BMPs can be divided into four groups: 1) BMP2/4; 2) BMP5, 6, 7, 8a, and 8b; 3) BMP9/10; 4) BMP12, 13 and 14 [38–40]. BMP1 is a unique protein that does not belong to the TGF-β superfamily [41,42]. The C-terminus of mature BMPs contains an active structural motif consisting of seven conserved cysteine amino acids [43]. BMP proteins are capable of binding to cell surface receptors to induce phosphorylation of the downstream substrates Smad1/5/8. The phosphorylated Smad1/5/8 (P-Smad1/5/8) associate with Smad4, forming a complex that then translocates into the nucleus to modulate downstream anti-fibrotic gene expression, such as ID1 and ID2 [35,44,45]. TGF-β1 exhibits pro-fibrotic roles by inducing myofibroblast differentiation and ECM production through activation of Smad2/3 [35,46]. An increasing number of studies have indicated that BMP proteins likely exhibit anti-fibrotic functions in the pathogenesis of fibrosis in lung and other organs through activation of Smad1/5/8 [9–12,47,48] (Fig. 1). In addition to inducing pro- or anti-fibrotic gene expression through Smad2/3 or Smad1/5/8 by TGF-β or BMPs, the BMP pathway, through separate mechanisms also competes with TGF-β signaling to protect against fibrogenesis [49–51]. Though the Smad1/5/8-mediated canonical pathway has been identified to play a critical role in the anti-fibrotic effect of BMPs, the non-canonical pathway of BMPs have not been well studied.

**Fig. 2.**
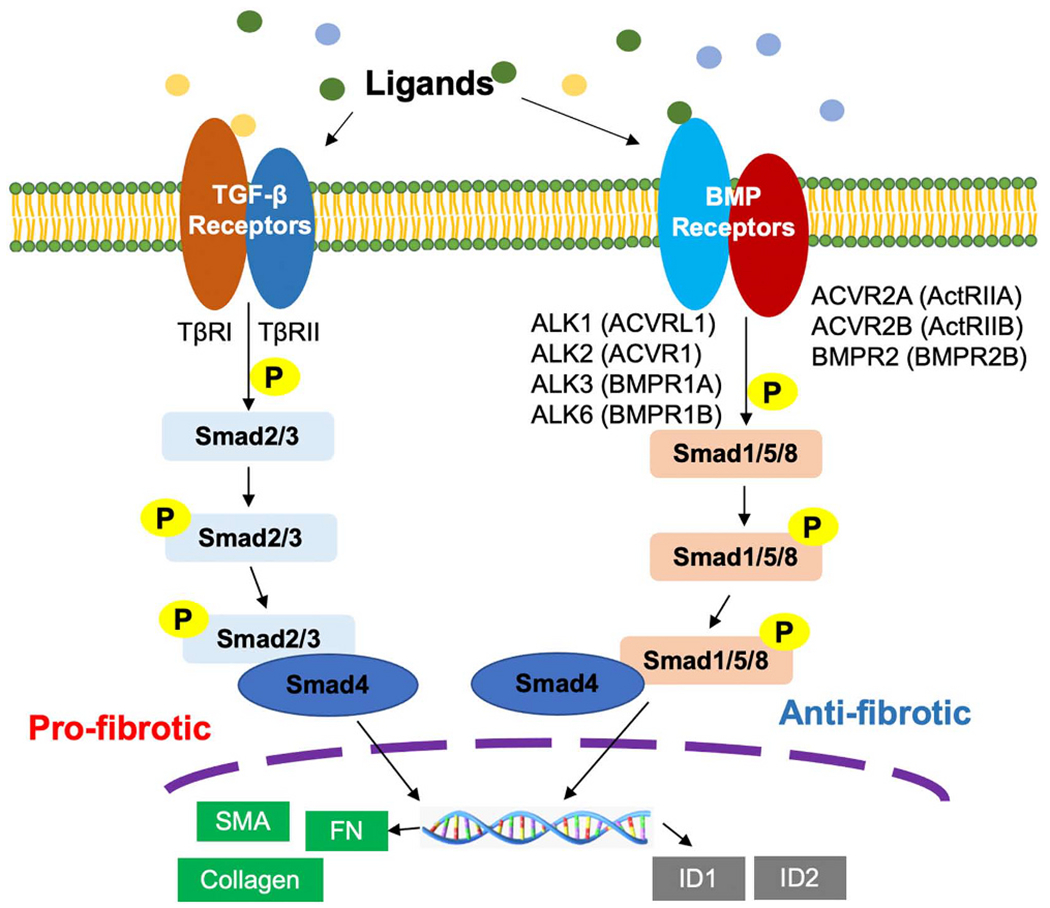
TGF-β and BMP receptors and their signaling pathways. BMP type I receptors have 4 subtypes including ALK1 (ACVRL1), ALK2 (ACVR1), ALK3 (BMPR1A) and ALK6 (BMPR1B); type II receptors consist of ACVR2A (ActRIIA), ACVR2B (ActRIIB) and BMPR2B (BMPR2). TGF-β signaling pathway plays a pro-fibrotic role to promote the α-smooth muscle actin (SMA), fibronectin (FN), and collagen expression; while BMP signaling pathway indicates the anti-fibrotic function.

**Fig. 3.**
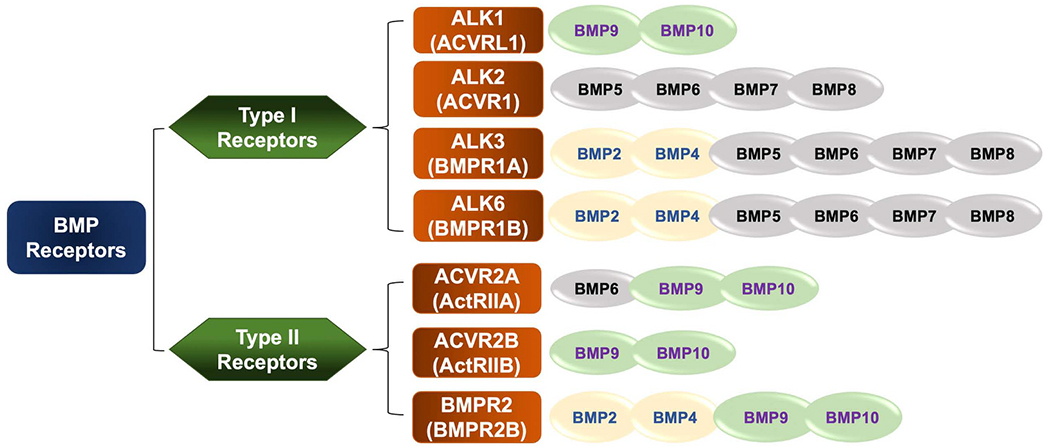
Binding affinities of various BMPs for BMP receptors. BMP ligands have different binding affinities for each type I and type II of BMP receptors.

There are two types of BMP receptors, type I and type II receptors that form hetero-tetrameric complex on the cell surface. ALK1 (ACVRL1), BMPR1A (ALK3), BMPR1B (ALK6), and ALK2 (ACVR1) are BMP type I receptors. Type II receptors have three subtypes including BMPR2, ACVR2A (ActRIIA), and ACVR2B (ActRIIB) [8,49–51] (Fig. 2). All of subtypes of BMP receptors are known to bind BMP ligands. BMPRs contain a short extracellular domain, a membrane-spanning domain, and an intracellular serine/threonine kinase domain [52]. The structures of ALK3 and ALK6 in the BMPR type I group are highly similar, while ALK2 is distinct from the others [44]. BMPR2 has constitutively protein kinase activity, and it contains a long C-terminal domain positioned next to the kinase domain [50]. It is generally believed that BMPs can bind BMPR1 and BMPR2 complex on the cell surface to initiate intracellular signaling [53]. The affinities of various BMP ligands for different BMP receptors are diverse. BMP2/4 bind to ALK3 and ALK6; the signaling of BMP5, 6, 7 and 8 is through ALK2, 3, and 6; BMP9/10 principally recognizes ALK1 [6,54,55] (Fig. 3). Type II receptors also exhibit various binding affinities to BMPs. For example, BMP2, 4, 9, and 10 bind to BMPR2; BMP6, 9, and 10 bind to ACVR2A; and BMP9 and BMP10 bind to ACVR2B (Fig. 3). BMPR2 can only be recognized by BMPs, while ACVR2A/2B can be activated by other ligands within the TGF-β family, such as activins [55,56]. Some BMPs not only signal through type I and type II BMP receptors, but also can induce downstream signaling through TGF-βRs.

## 5. Expression of BMPs and BMPR2 in PF

### 5.1. Altered expression of BMPs in IPF lungs

**Fig. 4.**
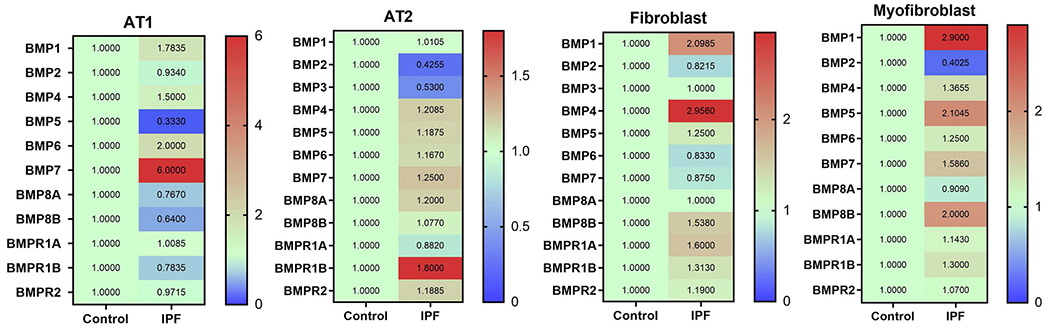
Changes of gene expression of BMP ligands and receptors in IPF lungs normalized with control health subjects. Data is re analyzed from http://ipfcellatlas.com/.

Single cell RNA-seq data has revealed genetic alternations of BMP ligands and receptors in different lung cell types from both normal and IPF patients (Fig. 4). There is a dramatic increase in gene expression of BMP1 in fibroblasts and myofibroblasts in IPF lungs compared to control subjects, but BMP1 has been shown to not be required for development of lung fibrosis in a murine model of PF [57]. Levels of BMP2, 3, 5, 6 and 7 are significantly altered in alveolar epithelial cells in IPF lungs. The expression of BMP2 and 3 exhibit 2-fold reduction in alveolar type 2 (AT2) cells in IPF patients, whereas gene expression of BMP7 is significantly increased in AT1 cells in IPF patients compared to normal subjects. Furthermore, the expression of BMP4 and BMP5 genes vary in fibroblasts and myofibroblasts in IPF lungs as well. BMP5 is increased 1-fold more in myofibroblasts in IPF lungs. BMP4 gene expression is up-regulated 2-fold in fibroblasts in the IPF lungs (Fig. 4); however, reduction of protein level of BMP4 in IPF lungs has been reported [47]. Guan et al. demonstrated that the protein level of BMP4 was down-regulated in lung tissues and fibroblasts from IPF patients and bleomycin-induced mice lung fibrosis. Thus, this raises a caution to investigators in the field that scRNA-seq data can be used to compare gene levels of BMPs, but the protein levels in IPF lungs should be confirmed by immunoblotting or immunohistology staining. The molecular regulation of BMPs has not been studied. Further investigation may focus on identifying transcriptional or suppression factors for BMPs, and examining DNA methylation of BMPs’ promoter and mRNA stability in the setting of PF.

### 5.2. Altered expression of BMPR2 in IPF lungs

It has been revealed that BMPR2 level is significantly downregulated in fibrotic area in IPF lungs compared to normal subjects [58–60]. This was confirmed in bleomycin-induced experimental lung fibrosis [58,59]. Consistently, the levels of p-Smad1/5/8, the downstream pathway of BMPR2, in IPF lungs were also found to be reduced [59]. BMPR2 protein has also been identified to be decreased in TGF-β1-treated rat lung fibroblasts [59,60]. Moreover, decreased BMPR2 signaling has been observed in endothelial cells (EC) and vascular smooth muscle cells (VSMCs) in fibrotic lungs in a rat model of experimental PF and in IPF patients [58,60]. BMPR2 is well known to be strongly associated with the pathogenesis of PAH. The symptoms and duration of PH and PF are tightly correlated. BMPR2 and p-Smad1/5/8 protein levels have been shown to be significantly reduced in lungs in IPF or IPF+PH patients compared to normal control lung tissues [18,58]. Few studies have been published investigating molecular regulation of BMPR2 expression in lungs. The effect of BMPs on BMPR2 expression has not been reported. Elevated IL-6 and STAT3 may correlate with the reduction of BMPR2 through upregulation of certain microRNAs (miRs) in the setting of lung fibrosis [58,61]. MiR-215–5p was shown to suppress BMPR2 expression in mouse lung epithelial (MLE) cells [62]. The molecular mechanisms of regulation of BMPR2 in PF remains unknown. BMPR2 protein stability has been shown to be regulated by ubiquitination in pulmonary artery endothelial and smooth muscle cells. Smurf1 E3 ligase targets BMPR2 for its ubiquitination and degradation [63,64]. The ubiquitination site, Smurf1 binding site on BMPR2, as well as whether Smurf1 regulation of BMPR2 stability in lung fibroblasts have not been revealed. Identification of Smurf1 binding site and a deubiquitinase for BMPR2 stabilization may be beneficial for development of small molecules to regulate BMPR2 protein stability.

## 6. The effects of BMPs and BMPRs signaling in PF

### 6.1. BMP4 & BMP7 protect against PF

Although BMP members have been revealed to play an anti-fibrotic function in several different organs [60,67–70], BMP4 and BMP7 are the most intensively studied in pulmonary fibrosis. Therefore, we will focus on BMP4 and BMP7 in lung fibrosis.

#### 6.1.1. The effects of BMP4 in PF

BMP4 is a key member of the BMP family that plays roles in embryonic development and bone formation [65,66]. A previous study has demonstrated that BMP4 highlights an important factor in cardiovascular diseases [67]. Recently, Peng’s group has discovered that inhalation of BMP4 into lungs can restore normal lung regeneration and improve lung function in a murine lung fibrosis model [68]. The potential therapeutic effect of BMP4 in lung fibrosis was confirmed by another recent study from Guan et al. [47]. The authors showed that overexpression of BMP4 with an AAV system reversed bleomyin-induced murine lung fibrosis and promoted lung fibrosis resolution. Importantly, they showed that BMP4 deficient mice were more susceptible to developing PF in mice. Mechanistically, BMP4 treatment suppresses TGF-β1-induced phosphorylation of Smad2/3, α-SMA expression, and ECM protein production in mouse lung fibroblasts [47]. This data was consistent from a study by Pegorier, T.K. et al. that showed that BMP4 attenuated TGF-β1-mediated MMP13 production in human lung fibroblasts [11]. In addition, gremlin, an antagonist of BMPs including BMP4, has been discovered to be greatly increased in IPF patients, as well as in the context of TGF-β1-treated lung epithelial cells. Gremlin enhanced the fibrotic response and reduced epithelial regeneration through inhibition of BMP4 in the lungs [69–71]. Further, Guan et al. revealed that BMP4 decreased oxidative damage in lung fibroblasts, promoted mitophagy, and restored mitochondrial dynamics in TGF-β1-stimulated lung myofibroblasts [47]. These observations suggest that the anti-fibrotic effects of BMP4 occurs both through attenuation of TGF-β1 pathways and independent modulation of TGF-β1 pathways. However, not all studies support the conclusion that BMP4 antagonizes TGF-β1 pathways. In Pegorier, S. et al.’s study, BMP4 attenuated TGF-β1-induced FN mRNA and protein levels, but had no effect on α-SMA levels in human lung fibroblasts [11], suggesting that BMP4 does not attenuate all the pro-fibrotic effects induced by TGF-β1. Although most studies support that BMP4 exhibits an anti-fibrotic role, an earlier study reported that BMP4 enhanced lung fibroblast differentiation to myofibroblasts [72]. Other studies reported that BMP4 induced EMT in lungs and pancreas, which may contribute to organ fibrosis [73]. The cause of the discrepancy of the effects of BMP4 is unclear. According to the published literatures, it is well accepted that BMP4 exerts antifibrotic effects in the lungs, particularly in lung fibroblasts. Its effect in lung epithelial cells needs more investigation. These findings may guide new research directions for the utilization of BMP4 in IPF therapy.

#### 6.1.2. The effect of BMP7 in PF

BMP7 has been reported to play an anti-fibrotic role in kidney, heart, and liver fibrosis through inhibiting TGF-β1-induced EMT and fibrotic responses in fibroblasts [9,74]. Zeisberg, M. et al. demonstrated that BMP7 promoted mesenchymal to epithelial transition (MET) in rental fibroblasts that facilities renal tissue repair after injury [75]. BMP7 not only induces phosphorylation of Smad1/5, but also suppresses TGF-β1-mediated Smad2/3 phosphorylation [9]. Phosphorylated Smad1/5 competes with phosphorylated Smad2/3 for binding to Smad4 [76]. Pegorier, S. et al. showed that BMP7 attenuated TGF-β1-induced α-SMA expression in human lung fibroblasts [11]. However, Knight’s team discovered that BMP7 could not protect against bleomycin-induced lung fibrosis [77]. Notably, this study did not examine the effect of BMP7 in lung fibroblasts. Additionally, the authors used human recombinant BMP7 in a murine model of PF, it is unclear whether human BMP7 exhibits the same effect as murine BMP7 in the *in vivo* study. Further, BMP7 has been shown to alleviate silica-induced pulmonary fibrosis in rats. BMP7 is capable of reducing expression of vimentin in the silica-induced lung fibrosis model and inhibiting EMT through suppressing TGF-β/Smad2/3 pathways [78,79]. Although the findings regarding the effects of BMP7 in fibrosis are still debatable, it remains a relatively well-recognized potential therapeutic approach to treat fibrotic diseases [80]. It is expected that further progress will be obtained using BMP7 combined with BMP4.

### 6.2. The therapeutic potential of BMPR2 in treating PF

Based on the reduction of BMPR2 in fibrotic lungs and anti-fibrotic effects exhibited by BMPs, it has been hypothesized that overexpression of BMPR2 may attenuate TGF-β1-mediated pathways and alleviate the severity of PF. This was examined in lung fibroblasts. Fukihara et al. showed that overexpression of BMPR2 with adenovirus suppressed the TGF-β1-induced p-Smad2/3 pathway. BMPR2 overexpression promoted the effect of BMP7 on inhibition of TGF-β-induced FN lung fibroblasts. Further, overexpression of BMPR2 reduced the phosphorylation of p38 MARK in lung fibroblasts [59]. Therefore, BMPR2 signaling is believed to suppress TGF-β signaling and fibroblast activation. The therapeutic potential of BMPR2 in treating PF needs to be evaluated in more studies using *in vivo* models of PF. Strategies to increase BMPR2 levels, such as upregulation of BMPR2 expression by AAV or increase in protein stability by decreasing its ubiquitination, may therefore have therapeutic potentials for treating PF.

## 7. Conclusions and perspectives

BMPs and BMPR2 in PF and PAH have attracted interests from researchers. BMP ligands, particularly BMP4/7, have been found to play roles in alleviation of PF. Previous research works on BMPR2 have been focused on PAH, but recently its function in PF has also been gained attention. Numerous studies have demonstrated that reduced expression of BMPR2 in fibrotic lungs exacerbates PF. The prevailing concept is that BMPR2 plays an anti-fibrotic role. Based on the symptomatic similarities and strong clinical correlation between PAH and PF, and it is believed that upregulation of BMPs and BMPR2 may provide new clinical therapeutic options for treating PF. Inhibitors of BMPs/BMP receptors have been developed and they are useful tools to investigate the molecular mechanisms of BMPs-mediated signaling and biological effects (Table 1) [81–101]; however, small molecules of BMP/BMPR2 activators and stabilizers are not commercially available. This review suggests a new direction for development of drugs to treat PF.

## Figures and Tables

**Table 1:** Inhibitors/antagonists of BMPs and BMP receptors.

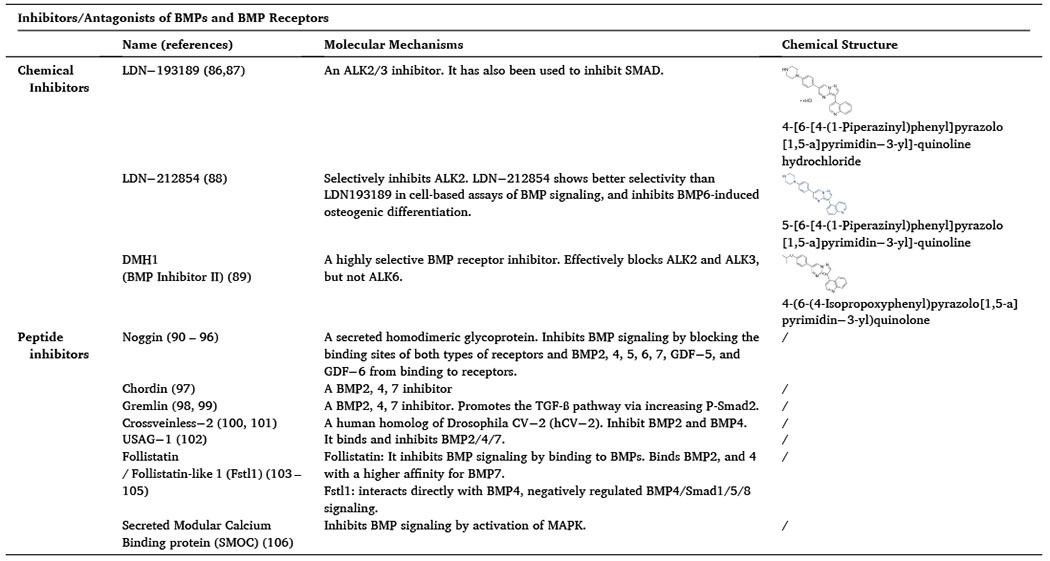

## Data Availability

No data was used for the research described in the article.

## References

[1] Wang Y, Zhang L, Wu GR, , MBD2 serves as a viable target against pulmonary fibrosis by inhibiting macrophage M2 program, Sci. Adv 7 (1) (2021). 10.1126/sciadv.abb6075 PMC7775789 33277324

[2] Zisman DA, Keane MP, Belperio JA, Strieter RM, Lynch JP 3rd. Pulmonary fibrosis, Methods Mol. Med 117 (2005) 3–44. 16130230 10.1385/1-59259-940-0:003 PMC7120641

[3] Luppi F, Kalluri M, Faverio P, Kreuter M, Ferrara G, Idiopathic pulmonary fibrosis beyond the lung: understanding disease mechanisms to improve diagnosis and management, Respir. Res 22 (1) (2021) 109. 33865386 10.1186/s12931-021-01711-1 PMC8052779

[4] Raghu G, Remy-Jardin M, Richeldi L, , Idiopathic pulmonary fibrosis (an update) and progressive pulmonary fibrosis in adults: an official ATS/ERS/JRS/ALAT clinical practice guideline, Am. J. Respir. Crit. Care Med 205 (9) (2022) e18–e47. 35486072 10.1164/rccm.202202-0399ST PMC9851481

[5] Urist MR, Strates BS, Bone morphogenetic protein, J. Dent. Res 50 (6) (1971) 1392–1406. 4943222 10.1177/00220345710500060601

[6] Hinck AP, Mueller TD, Springer TA, Structural Biology and Evolution of the TGF-beta Family. Cold Spring Harb. Perspect. Biol 8 (12) (2016). 10.1101/cshperspect.a022103 PMC5131774 27638177

[7] Yadin D, Knaus P, Mueller TD, Structural insights into BMP receptors: Specificity, activation and inhibition, Cytokine Growth Factor Rev. 27 (2016) 13–34. 26690041 10.1016/j.cytogfr.2015.11.005

[8] Yamashita H, ten Dijke P, Huylebroeck D, , Osteogenic protein-1 binds to activin type II receptors and induces certain activin-like effects, J. Cell Biol 130 (1) (1995) 217–226. 7790373 10.1083/jcb.130.1.217 PMC2120513

[9] Zeisberg M, Hanai J, Sugimoto H, , BMP-7 counteracts TGF-beta1-induced epithelial-to-mesenchymal transition and reverses chronic renal injury, Nat. Med 9 (7) (2003) 964–968. 12808448 10.1038/nm888

[10] Wang S, Hirschberg R, BMP7 antagonizes TGF-beta -dependent fibrogenesis in mesangial cells, Am. J. Physiol. Ren. Physiol 284 (5) (2003) F1006–F1013. 10.1152/ajprenal.00382.2002 12676736

[11] Pegorier S, Campbell GA, Kay AB, Lloyd CM, Bone morphogenetic protein (BMP)-4 and BMP-7 regulate differentially transforming growth factor (TGF)-beta1 in normal human lung fibroblasts (NHLF), Respir. Res 11 (1) (2010) 85. 10.1186/1465-9921-11-85 PMC2898775 20573231

[12] Weiskirchen R, Meurer SK, BMP-7 counteracting TGF-beta1 activities in organ fibrosis, Front. Biosci. (Landmark Ed.) 18 (4) (2013) 1407–1434. 23747893 10.2741/4189

[13] Pousada G, Lupo V, Castro-Sanchez S, , Molecular and functional characterization of the BMPR2 gene in Pulmonary Arterial Hypertension, Sci. Rep 7 (1) (2017) 1923. 28507310 10.1038/s41598-017-02074-8 PMC5432510

[14] Morrell NW, Aldred MA, Chung WK, , Genetics and genomics of pulmonary arterial hypertension, Eur. Respir. J 53 (1) (2019). 10.1183/13993003.01899-2018 PMC6351337 30545973

[15] Hemnes AR, Humbert M, Pathobiology of pulmonary arterial hypertension: understanding the roads less travelled, Eur. Respir. Rev 26 (146) (2017). 10.1183/16000617.0093-2017 PMC9488187 29263173

[16] Atkinson C, Stewart S, Upton PD, , Primary pulmonary hypertension is associated with reduced pulmonary vascular expression of type II bone morphogenetic protein receptor, Circulation 105 (14) (2002) 1672–1678. 11940546 10.1161/01.cir.0000012754.72951.3d

[17] Machado RD, Eickelberg O, Elliott CG, , Genetics and genomics of pulmonary arterial hypertension, J. Am. Coll. Cardiol 54 (1 Suppl) (2009) S32–S42. 19555857 10.1016/j.jacc.2009.04.015 PMC3725550

[18] Bryant AJ, Robinson LJ, Moore CS, , Expression of mutant bone morphogenetic protein receptor II worsens pulmonary hypertension secondary to pulmonary fibrosis, Pulm. Circ 5 (4) (2015) 681–690. 26697175 10.1086/683811 PMC4671742

[19] Podolanczuk AJ, Thomson CC, Remy-Jardin M, , Idiopathic pulmonary fibrosis: state of the art for 2023, Eur. Respir. J 61 (4) (2023). 10.1183/13993003.00957-2022 36702498

[20] Darby IA, Laverdet B, Bonte F, Desmouliere A, Fibroblasts and myofibroblasts in wound healing, Clin. Cosmet. Invest. Dermatol 7 (2014) 301–311. 10.2147/CCID.S50046 PMC4226391 25395868

[21] Iredale JP, Benyon RC, Pickering J, , Mechanisms of spontaneous resolution of rat liver fibrosis. Hepatic stellate cell apoptosis and reduced hepatic expression of metalloproteinase inhibitors, J. Clin. Invest 102 (3) (1998) 538–549. 9691091 10.1172/JCI1018 PMC508915

[22] Desmouliere A, Redard M, Darby I, Gabbiani G, Apoptosis mediates the decrease in cellularity during the transition between granulation tissue and scar, Am. J. Pathol 146 (1) (1995) 56–66. 7856739 PMC1870783

[23] Redente EF, Chakraborty S, Sajuthi S, , Loss of Fas signaling in fibroblasts impairs homeostatic fibrosis resolution and promotes persistent pulmonary fibrosis, JCI Insight 6 (1) (2020). 10.1172/jci.insight.141618 PMC7821600 33290280

[24] Kisseleva T, Cong M, Paik Y, , Myofibroblasts revert to an inactive phenotype during regression of liver fibrosis, Proc. Natl. Acad. Sci. USA 109 (24) (2012) 9448–9453. 22566629 10.1073/pnas.1201840109 PMC3386114

[25] Jun JI, Lau LF, Resolution of organ fibrosis, J. Clin. Invest 128 (1) (2018) 97–107. 29293097 10.1172/JCI93563 PMC5749507

[26] Stramer BM, Mori R, Martin P, The inflammation-fibrosis link? A Jekyll and Hyde role for blood cells during wound repair, J. Invest Dermatol 127 (5) (2007) 1009–1017. 17435786 10.1038/sj.jid.5700811

[27] Gurtner GC, Werner S, Barrandon Y, Longaker MT, Wound repair and regeneration, Nature 453 (7193) (2008) 314–321. 18480812 10.1038/nature07039

[28] Moore MW, Herzog EL, Regulation and relevance of myofibroblast responses in idiopathic pulmonary fibrosis, Curr. Pathobiol. Rep 1 (3) (2013) 199–208. 25705577 10.1007/s40139-013-0017-8 PMC4334480

[29] Iwano M, Plieth D, Danoff TM, Xue C, Okada H, Neilson EG, Evidence that fibroblasts derive from epithelium during tissue fibrosis, J. Clin. Invest 110 (3) (2002) 341–350. 12163453 10.1172/JCI15518 PMC151091

[30] Shaw TJ, Rognoni E, Dissecting fibroblast heterogeneity in health and fibrotic disease, Curr. Rheuma Rep 22 (8) (2020) 33. 10.1007/s11926-020-00903-w PMC7305072 32562113

[31] Roman J, Fibroblasts-warriors at the intersection of wound healing and disrepair, Biomolecules 13 (6) (2023). 10.3390/biom13060945 PMC10296409 37371525

[32] Selman M, Pardo A, When things go wrong: exploring possible mechanisms driving the progressive fibrosis phenotype in interstitial lung diseases, Eur. Respir. J 58 (3) (2021). 10.1183/13993003.04507-2020 33542060

[33] Liu S, Guo J, Cheng X, , Molecular evolution of transforming growth factor-beta (TGF-beta) gene family and the functional characterization of lamprey TGF-beta2, Front. Immunol 13 (2022) 836226. 35309318 10.3389/fimmu.2022.836226 PMC8931421

[34] Morikawa M, Derynck R, Miyazono K, TGF-beta and the TGF-beta family: context-dependent roles in cell and tissue physiology, Cold Spring Harb. Perspect. Biol 8 (5) (2016). 10.1101/cshperspect.a021873 PMC4852809 27141051

[35] Saito A, Horie M, Nagase T, TGF-beta Signaling in Lung Health and Disease, Int. J. Mol. Sci 19 (8) (2018). 10.3390/ijms19082460 PMC6121238 30127261

[36] Cheng H, Jiang W, Phillips FM, , Osteogenic activity of the fourteen types of human bone morphogenetic proteins (BMPs), J. Bone Jt. Surg. Am 85 (8) (2003) 1544–1552. 10.2106/00004623-200308000-00017 12925636

[37] Chen G, Deng C, Li YP, TGF-beta and BMP signaling in osteoblast differentiation and bone formation, Int. J. Biol. Sci 8 (2) (2012) 272–288. 22298955 10.7150/ijbs.2929 PMC3269610

[38] Carreira AC, Lojudice FH, Halcsik E, Navarro RD, Sogayar MC, Granjeiro JM, Bone morphogenetic proteins: facts, challenges, and future perspectives, J. Dent. Res 93 (4) (2014) 335–345. 24389809 10.1177/0022034513518561

[39] von Bubnoff A, Cho KW, Intracellular BMP signaling regulation in vertebrates: pathway or network? Dev. Biol 239 (1) (2001) 1–14. 11784015 10.1006/dbio.2001.0388

[40] Nickel J, Mueller TD, Specification of BMP signaling, Cells 8 (12) (2019). 10.3390/cells8121579 PMC6953019 31817503

[41] Bond JS, Beynon RJ, The astacin family of metalloendopeptidases, Protein Sci. 4 (7) (1995) 1247–1261. 7670368 10.1002/pro.5560040701 PMC2143163

[42] Scott IC, Imamura Y, Pappano WN, , Bone morphogenetic protein-1 processes probiglycan, J. Biol. Chem 275 (39) (2000) 30504–30511. 10896944 10.1074/jbc.M004846200

[43] Bessa PC, Casal M, Reis RL, Bone morphogenetic proteins in tissue engineering: the road from the laboratory to the clinic, part I (basic concepts), J. Tissue Eng. Regen. Med 2 (1) (2008) 1–13. 18293427 10.1002/term.63

[44] Miyazono K, Maeda S, Imamura T, BMP receptor signaling: transcriptional targets, regulation of signals, and signaling cross-talk, Cytokine Growth Factor Rev. 16 (3) (2005) 251–263. 15871923 10.1016/j.cytogfr.2005.01.009

[45] Wu M, Chen G, Li YP, TGF-beta and BMP signaling in osteoblast, skeletal development, and bone formation, homeostasis and disease, Bone Res. 4 (2016) 16009. 27563484 10.1038/boneres.2016.9 PMC4985055

[46] Yue X, Shan B, Lasky JA, TGF-beta: titan of lung fibrogenesis, Curr. Enzym Inhib 6 (2) (2010). 10.2174/10067 PMC3812949 24187529

[47] Guan R, Yuan L, Li J, , Bone morphogenetic protein 4 inhibits pulmonary fibrosis by modulating cellular senescence and mitophagy in lung fibroblasts, Eur. Respir. J 60 (6) (2022). 10.1183/13993003.02307-2021 PMC9808813 35777761

[48] Morine KJ, Qiao X, York S, , Bone morphogenetic protein 9 reduces cardiac fibrosis and improves cardiac function in heart failure, Circulation 138 (5) (2018) 513–526. 29487140 10.1161/CIRCULATIONAHA.117.031635 PMC6111008

[49] Koenig BB, Cook JS, Wolsing DH, , Characterization and cloning of a receptor for BMP-2 and BMP-4 from NIH 3T3 cells, Mol. Cell Biol 14 (9) (1994) 5961–5974. 8065329 10.1128/mcb.14.9.5961 PMC359122

[50] Rosenzweig BL, Imamura T, Okadome T, , Cloning and characterization of a human type II receptor for bone morphogenetic proteins, Proc. Natl. Acad. Sci. USA 92 (17) (1995) 7632–7636. 7644468 10.1073/pnas.92.17.7632 PMC41199

[51] Rahman MS, Akhtar N, Jamil HM, Banik RS, Asaduzzaman SM, TGF-beta/BMP signaling and other molecular events: regulation of osteoblastogenesis and bone formation, Bone Res. 3 (2015) 15005. 26273537 10.1038/boneres.2015.5 PMC4472151

[52] Rao SM, Ugale GM, Warad SB, Bone morphogenetic proteins: periodontal regeneration, N. Am. J. Med. Sci 5 (3) (2013) 161–168. 23626951 10.4103/1947-2714.109175 PMC3632019

[53] Allendorph GP, Vale WW, Choe S, Structure of the ternary signaling complex of a TGF-beta superfamily member. Proc. Natl. Acad. Sci. USA 103 (20) (2006) 7643–7648. 16672363 10.1073/pnas.0602558103 PMC1456805

[54] Ebisawa T, Tada K, Kitajima I, , Characterization of bone morphogenetic protein-6 signaling pathways in osteoblast differentiation, J. Cell Sci 112 (Pt 20) (1999) 3519–3527. 10504300 10.1242/jcs.112.20.3519

[55] Kim MJ, Park SY, Chang HR, , Clinical significance linked to functional defects in bone morphogenetic protein type 2 receptor, BMPR2, BMB Rep. 50 (6) (2017) 308–317. 28391780 10.5483/BMBRep.2017.50.6.059 PMC5498141

[56] Chu KY, Malik A, Thamilselvan V, Martinez-Hackert E, Type II BMP and activin receptors BMPR2 and ACVR2A share a conserved mode of growth factor recognition, J. Biol. Chem 298 (7) (2022) 102076. 35643319 10.1016/j.jbc.2022.102076 PMC9234707

[57] Ma HY, N’Diaye EN, Caplazi P, , BMP1 is not required for lung fibrosis in mice, Sci. Rep 12 (1) (2022) 5466. 35361882 10.1038/s41598-022-09557-3 PMC8971496

[58] Chen NY, Luo DCS,F, , Macrophage bone morphogenic protein receptor 2 depletion in idiopathic pulmonary fibrosis and Group III pulmonary hypertension, Am. J. Physiol. Lung Cell Mol. Physiol 311 (2) (2016) L238–L254. 27317687 10.1152/ajplung.00142.2016 PMC6425517

[59] Fukihara J, Maiolo S, Kovac J, , Overexpression of bone morphogenetic protein receptor type 2 suppresses transforming growth factor beta-induced profibrotic responses in lung fibroblasts, Exp. Lung Res 48 (1) (2022) 35–51. 35037801 10.1080/01902148.2021.2024301

[60] Yanagihara T, Tsubouchi K, Zhou Q, , Vascular-parenchymal cross-talk promotes lung fibrosis through BMPR2 signaling, Am. J. Respir. Crit. Care Med 207 (11) (2023) 1498–1514. 36917778 10.1164/rccm.202109-2174OC

[61] Wong S, Botelho FM, Rodrigues RM, Richards CD, Oncostatin M overexpression induces matrix deposition, STAT3 activation, and SMAD1 Dysregulation in lungs of fibrosis-resistant BALB/c mice, Lab Invest. 94 (9) (2014) 1003–1016. 24933422 10.1038/labinvest.2014.81

[62] Huang J, Cao Y, Li X, Yu F, Han X, E2F1 regulates miR-215-5p to aggravate paraquat-induced pulmonary fibrosis via repressing BMPR2 expression, Toxicol. Res. (Camb.) 11 (6) (2022) 940–950. 36569483 10.1093/toxres/tfac071 PMC9773066

[63] Murakami K, Etlinger JD, Role of SMURF1 ubiquitin ligase in BMP receptor trafficking and signaling, Cell Signal 54 (2019) 139–149. 30395943 10.1016/j.cellsig.2018.10.015

[64] Guo L, Wang R, Zhang K, , A PINCH-1-Smurf1 signaling axis mediates mechano-regulation of BMPR2 and stem cell differentiation, J. Cell Biol 218 (11) (2019) 3773–3794. 31578224 10.1083/jcb.201902022 PMC6829670

[65] Dong X, Mao Y, Gao P, The role of bone morphogenetic protein 4 in lung diseases, Curr. Mol. Med 23 (4) (2023) 324–331. 36883260 10.2174/1566524022666220428110906

[66] Xie Z, Zhou G, Zhang M, , Recent developments on BMPs and their antagonists in inflammatory bowel diseases, Cell Death Discov. 9 (1) (2023) 210. 37391444 10.1038/s41420-023-01520-z PMC10313712

[67] Guo WT, Dong DL, Bone morphogenetic protein-4: a novel therapeutic target for pathological cardiac hypertrophy/heart failure, Heart Fail Rev. 19 (6) (2014) 781–788. 24736806 10.1007/s10741-014-9429-8

[68] Cassandras M, Wang C, Kathiriya J, , Gli1(+) mesenchymal stromal cells form a pathological niche to promote airway progenitor metaplasia in the fibrotic lung. Nat. Cell Biol 22 (11) (2020) 1295–1306. 33046884 10.1038/s41556-020-00591-9 PMC7642162

[69] Koli K, Myllarniemi M, Vuorinen K, , Bone morphogenetic protein-4 inhibitor gremlin is overexpressed in idiopathic pulmonary fibrosis, Am. J. Pathol 169 (1) (2006) 61–71. 16816361 10.2353/ajpath.2006.051263 PMC1698771

[70] Myllarniemi M, Lindholm P, Ryynanen MJ, , Gremlin-mediated decrease in bone morphogenetic protein signaling promotes pulmonary fibrosis, Am. J. Respir. Crit. Care Med 177 (3) (2008) 321–329. 17975199 10.1164/rccm.200706-945OC PMC2218851

[71] Myllarniemi M, Vuorinen K, Pulkkinen V, , Gremlin localization and expression levels partially differentiate idiopathic interstitial pneumonia severity and subtype, J. Pathol 214 (4) (2008) 456–463. 18072275 10.1002/path.2300

[72] Jeffery TK, Upton PD, Trembath RC, Morrell NW, BMP4 inhibits proliferation and promotes myocyte differentiation of lung fibroblasts via Smad1 and JNK pathways, Am. J. Physiol. Lung Cell Mol. Physiol 288 (2) (2005) L370–L378. 15516492 10.1152/ajplung.00242.2004

[73] Molloy EL, Adams A, Moore JB, , BMP4 induces an epithelial-mesenchymal transition-like response in adult airway epithelial cells, Growth Factors 26 (1) (2008) 12–22. 18365875 10.1080/08977190801987166

[74] Gonzalez EA, Lund RJ, Martin KJ, , Treatment of a murine model of high-turnover renal osteodystrophy by exogenous BMP-7, Kidney Int. 61 (4) (2002) 1322–1331. 11918739 10.1046/j.1523-1755.2002.00258.x

[75] Zeisberg M, Shah AA, Kalluri R, Bone morphogenic protein-7 induces mesenchymal to epithelial transition in adult renal fibroblasts and facilitates regeneration of injured kidney, J. Biol. Chem 280 (9) (2005) 8094–8100. 15591043 10.1074/jbc.M413102200

[76] Feng L, Cook B, Tsai SY, , Discovery of a small-molecule BMP sensitizer for human embryonic stem cell differentiation, Cell Rep. 15 (9) (2016) 2063–2075. 27210748 10.1016/j.celrep.2016.04.066 PMC4889468

[77] Murray LA, Hackett TL, Warner SM, , BMP-7 does not protect against bleomycin-induced lung or skin fibrosis, PLoS One 3 (12) (2008) e4039. 19112509 10.1371/journal.pone.0004039 PMC2603595

[78] Yang G, Zhu Z, Wang Y, Gao A, Niu P, Tian L, Bone morphogenetic protein-7 inhibits silica-induced pulmonary fibrosis in rats, Toxicol. Lett 220 (2) (2013) 103–108. 23639248 10.1016/j.toxlet.2013.04.017

[79] Liang D, An G, Zhu Z, , The protective effects of bone morphogenetic protein-7 against epithelial injury and matrix metalloproteases upregulation induced by silica in vitro, Hum. Exp. Toxicol 36 (9) (2017) 892–900. 28838258 10.1177/0960327116674527

[80] McVicker BL, Bennett RG, Novel anti-fibrotic therapies, Front. Pharm 8 (2017) 318. 10.3389/fphar.2017.00318 PMC5449464 28620300

[81] Boergermann JH, Kopf J, Yu PB, Knaus P, Dorsomorphin and LDN-193189 inhibit BMP-mediated Smad, p38 and Akt signalling in C2C12 cells, Int. J. Biochem. Cell Biol 42 (11) (2010) 1802–1807. 20691279 10.1016/j.biocel.2010.07.018 PMC6164168

[82] Cuny GD, Yu PB, Laha JK, , Structure-activity relationship study of bone morphogenetic protein (BMP) signaling inhibitors, Bioorg. Med. Chem. Lett 18 (15) (2008) 4388–4392. 18621530 10.1016/j.bmcl.2008.06.052 PMC2570262

[83] Williams E, Bullock AN, Structural basis for the potent and selective binding of LDN-212854 to the BMP receptor kinase ALK2, Bone 109 (2018) 251–258. 28918311 10.1016/j.bone.2017.09.004 PMC5871398

[84] Neely MD, Litt MJ, Tidball AM, , DMH1, a highly selective small molecule BMP inhibitor promotes neurogenesis of hiPSCs: comparison of PAX6 and SOX1 expression during neural induction, ACS Chem. Neurosci 3 (6) (2012) 482–491. 22860217 10.1021/cn300029t PMC3400384

[85] McMahon JA, Takada S, Zimmerman LB, Fan CM, Harland RM, McMahon AP, Noggin-mediated antagonism of BMP signaling is required for growth and patterning of the neural tube and somite, Genes Dev. 12 (10) (1998) 1438–1452. 9585504 10.1101/gad.12.10.1438 PMC316831

[86] Groppe J, Greenwald J, Wiater E, , Structural basis of BMP signalling inhibition by the cystine knot protein Noggin, Nature 420 (6916) (2002) 636–642. 12478285 10.1038/nature01245

[87] Groppe J, Greenwald J, Wiater E, , Structural basis of BMP signaling inhibition by Noggin, a novel twelve-membered cystine knot protein, J. Bone Jt. Surg. Am 85-A (Suppl 3) (2003) 52–58. 10.2106/00004623-200300003-00010 12925610

[88] Beck HN, Drahushuk K, Jacoby DB, Higgins D, Lein PJ, Bone morphogenetic protein-5 (BMP-5) promotes dendritic growth in cultured sympathetic neurons, BMC Neurosci. 2 (2001) 12. 11580864 10.1186/1471-2202-2-12 PMC56999

[89] Haudenschild DR, Palmer SM, Moseley TA, You Z, Reddi AH, Bone morphogenetic protein (BMP)-6 signaling and BMP antagonist noggin in prostate cancer, Cancer Res. 64 (22) (2004) 8276–8284. 15548695 10.1158/0008-5472.CAN-04-2251

[90] Merino R, Macias D, Ganan Y, , Expression and function of Gdf-5 during digit skeletogenesis in the embryonic chick leg bud, Dev. Biol 206 (1) (1999) 33–45. 9918693 10.1006/dbio.1998.9129

[91] Chang C, Hemmati-Brivanlou A, Xenopus GDF6, a new antagonist of noggin and a partner of BMPs, Development 126 (15) (1999) 3347–3357. 10393114 10.1242/dev.126.15.3347

[92] Reversade B, De Robertis EM, Regulation of ADMP and BMP2/4/7 at opposite embryonic poles generates a self-regulating morphogenetic field, Cell 123 (6) (2005) 1147–1160. 16360041 10.1016/j.cell.2005.08.047 PMC2292129

[93] Merino R, Rodriguez-Leon J, Macias D, Ganan Y, Economides AN, Hurle JM, The BMP antagonist Gremlin regulates outgrowth, chondrogenesis and programmed cell death in the developing limb, Development 126 (23) (1999) 5515–5522. 10556075 10.1242/dev.126.23.5515

[94] Sethi A, Jain A, Zode GS, Wordinger RJ, Clark AF, Role of TGFbeta/Smad signaling in gremlin induction of human trabecular meshwork extracellular matrix proteins, Invest. Ophthalmol. Vis. Sci 52 (8) (2011) 5251–5259. 21642622 10.1167/iovs.11-7587 PMC3176052

[95] Binnerts ME, Wen X, Cante-Barrett K, , Human Crossveinless-2 is a novel inhibitor of bone morphogenetic proteins, Biochem. Biophys. Res. Commun 315 (2) (2004) 272–280. 14766204 10.1016/j.bbrc.2004.01.048

[96] Ambrosio AL, Taelman VF, Lee HX, Metzinger CA, Coffinier C, De Robertis EM, Crossveinless-2 Is a BMP feedback inhibitor that binds Chordin/BMP to regulate Xenopus embryonic patterning, Dev. Cell 15 (2) (2008) 248–260. 18694564 10.1016/j.devcel.2008.06.013 PMC2581521

[97] Yanagita M, Oka M, Watabe T, , USAG-1: a bone morphogenetic protein antagonist abundantly expressed in the kidney, Biochem. Biophys. Res. Commun 316 (2) (2004) 490–500. 15020244 10.1016/j.bbrc.2004.02.075

[98] Pierre A, Pisselet C, Monget P, Monniaux D, Fabre S, Testing the antagonistic effect of follistatin on BMP family members in ovine granulosa cells, Reprod. Nutr. Dev 45 (4) (2005) 419–425. 16045890 10.1051/rnd:2005031

[99] Amthor H, Christ B, Rashid-Doubell F, Kemp CF, Lang E, Patel K, Follistatin regulates bone morphogenetic protein-7 (BMP-7) activity to stimulate embryonic muscle growth, Dev. Biol 243 (1) (2002) 115–127. 11846481 10.1006/dbio.2001.0555

[100] Geng Y, Dong Y, Yu M, , Follistatin-like 1 (Fstl1) is a bone morphogenetic protein (BMP) 4 signaling antagonist in controlling mouse lung development, Proc. Natl. Acad. Sci. USA 108 (17) (2011) 7058–7063. 21482757 10.1073/pnas.1007293108 PMC3084141

[101] Thomas JT, Canelos P, Luyten FP, Moos M Jr., Xenopus SMOC-1 Inhibits bone morphogenetic protein signaling downstream of receptor binding and is essential for postgastrulation development in Xenopus, J. Biol. Chem 284 (28) (2009) 18994–19005. 19414592 10.1074/jbc.M807759200 PMC2707235

